# Gene expression analyses in Atlantic salmon challenged with infectious salmon anemia virus reveal differences between individuals with early, intermediate and late mortality

**DOI:** 10.1186/1471-2164-9-179

**Published:** 2008-04-18

**Authors:** Sven Martin Jørgensen, Sergey Afanasyev, Aleksei Krasnov

**Affiliations:** 1 Nofima Marin AS, PO Box 5010, 1432 Ås, Norway; 2 Sechenov Institute of Evolutionary Physiology and Biochemistry, M. Toreza av. 44, Peterburg, 194223, Russia

## Abstract

**Background:**

Infectious salmon anemia virus (ISAV) causes a multisystemic disease responsible for severe losses in salmon aquaculture. Better understanding of factors that explain variations in resistance between individuals and families is essential for development of strategies for disease control. To approach this, we compared global gene expression using microarrays in fish dying early and late in the time course following infection from a highly pathogenic ISAV.

**Results:**

Tissues (gill, heart, liver and spleen) from infected Atlantic salmon (cohabitation, ISAV Glesvaer 2/90 isolate) were collected from three stages over the time course of the experiment; early (EM, 0–10% cumulative mortality (CM), 21–25 days post-infection (DPI)), intermediate (IM, 35–55% CM, 28–31 DPI) and late (LM, 75–85% CM, 37–48 DPI) mortality. Viral loads were equal in EM and IM but dropped markedly in LM fish. Gene expression analyses using a 1.8 K salmonid fish cDNA microarray (SFA2.0) and real-time qPCR revealed a large group of genes highly up-regulated across tissues in EM, which were mainly implicated in innate antiviral responses and cellular stress. Despite equal levels of MHC class I in EM and LM, increase of splenic and cardiac expression of immunoglobulin-like genes was found only in LM while a suite of adaptive immunity markers were activated already in IM. The hepatic responses to ISAV were characterized by difference between EM and LM in expression of chaperones and genes involved in eicosanoid metabolism. To develop classification of high and low resistance phenotypes based on a small number of genes, we processed results from qPCR analyses of liver using a linear discriminant analysis. Four genes (5-lipoxygenase activating protein, cytochrome P450 2K4-1, galectin-9 and annexin A1) were sufficient for correct assignment of individuals to EM, LM and uninfected groups, while IM was inseparable from EM. Three of four prognostic markers are involved in metabolism of inflammatory regulators.

**Conclusion:**

This study adds to the understanding of molecular determinants for resistance to acute ISAV infection. The most susceptible individuals were characterized by high viral replication and dramatic activation of innate immune responses, which did not provide protection. The ability to endure high levels of infection for sustained periods could be associated with lower inflammatory responses while subsequent protection and viral clearance was most likely conferred by activation of adaptive immunity.

## Background

Infectious salmon anemia has since the early 1990's been one of the most dangerous viral diseases threatening Atlantic salmon aquaculture industry. The infectious salmon anemia virus (ISAV) is a negative single-stranded RNA virus assigned to the genus *Isavirus *within the family *Orthomyxoviridae*. It mainly infects endothelial cells and leukocytes and causes a multisystemic disease characterized by high mortality with exophthalmia, pale gills, ascites, hemorrhagic liver necrosis, renal interstitial hemorrhage and tubular nephrosis (reviewed in [1,2]). Several ISAV strains with different pathogenicity are known and were recently categorized according to the genotype in the high polymorphic region of the hemagglutinin-esterase gene and the ability to induce acute versus protracted disease [3,4]. The fact that the virus' receptor-destroying enzyme (RDE) lacks activity in Atlantic salmon is thought to have an important role in mediating the severe clinical manifestations of infection (e.g. hemagglutination and anemia) [5,6]. Nonetheless, host factors must make a significant contribution to the outcome of infection since high variation in susceptibility and resistance to ISAV has been observed. Survival during natural and experimental infections by any particular virus isolate can range from 0–100% and varies significantly between families (R. Stigum Olsen, pers. comm.). The presence of a genetic component for disease resistance is strong evidence for the existence of protective antiviral responses, however potential molecular mechanisms that may account for protection against ISAV are unknown. Association between specific MHCI alleles and survival has been shown [7], which imply that a collection of multiple host factors may explain disease resistance. Although far from completely resolved, it seems that type 1 interferon (IFN) does not confer antiviral protection; this might be related to virus-antagonistic mechanisms [8,9]. There is also evidence that humoral immunity mounted by antibody responses is less important for survival [4]. Early studies also suggested that initial activation of T cells, but not B cells, and suppression of immune function caused by leukopenia is a general result of infection independent of anemia [10]. Collectively, these results imply that optimal constellation of multiple host factors are explaining disease resistance. This is also supported by the fact that low progression is achieved when breeding for ISAV resistance (R. Stigum Olsen, pers. comm.) and that only weak effect quantitative trait loci (QTL) for this trait have been found (T. Moen, pers. comm.).

In addition to the scientific interest, comparative studies of salmon with high and low resistance are of great importance for aquaculture. At present selective breeding and vaccination are key approaches to disease control, which employ respectively the innate and acquired protective mechanisms. Innate defence involves generalized antiviral responses, such as IFN and IFN-independent proteins and pathogen-specific antiviral factors referred to as restriction factors [11,12]. Adaptive immunity in turn includes humoral and cellular components which interact with a complex network of immune factors. Assessment of the relative roles of these mechanisms is vital for the development of efficient disease control strategies. Furthermore, today's selective breeding for disease resistance is hampered by a lack of predictive methods. Pathogen challenge enables identification of the most resistant individuals; however, infected fish may become carriers of pathogens and therefore cannot be returned to the farming environment. Thus, the development of methods for the prediction of resistance requires a better understanding of the protective host mechanisms.

To obtain a more comprehensive understanding and elucidation of important molecular determinants for survival towards infectious salmon anemia disease we have compared differences in global gene expression in resistant and susceptible fish using microarrray technology. Survival time is commonly used as an estimate of susceptibility and resistance in pathogen challenge tests. Atlantic salmon were infected with a common acute ISAV isolate by cohabitance, which mimics the natural mode of virus transfer. Tissues from fish within early (EM), intermediate (IM) and late mortality (LM) groups were sampled and viral loads were determined by real-time qPCR. We used a high-density salmonid fish cDNA microarray (SFA2.0 immunochip) designed for studies of responses to pathogens and stressors (GEO GPL1212) [13,14]. In comparison with previous versions, the updated 1.8 K platform (GEO GPL6154) has a substantially improved coverage of immune genes. The microarray analyses were conducted using EM and LM fish, and since the roles of tissues were not known we began with individual-pooled samples (n = 6) of gill, heart, spleen and liver. Next, individual analysis on liver samples (n = 6) was performed to find prognostic markers, since liver had the highest number of differentially expressed genes. The results of microarray analyses indicated possible molecular determinants of resistance, and to corroborate these conclusions, real-time qPCR analysis of genes selected by their functional roles and expression profiles were performed on individual samples (n = 4/n = 6). For this analysis we also included a number of genes being markers of adaptive immunity that were not present on the microarray platform. The development of predictors began with individual microarray comparisons using the liver tissue. Given high costs of microarray analyses it is customary to develop tests based on real-time qPCR trying to minimize the numbers of prognostic genes. We analyzed expression of the candidate genes and used linear discriminant analysis for the classification of samples.

## Results

### Experimental infection

**Figure 1:**
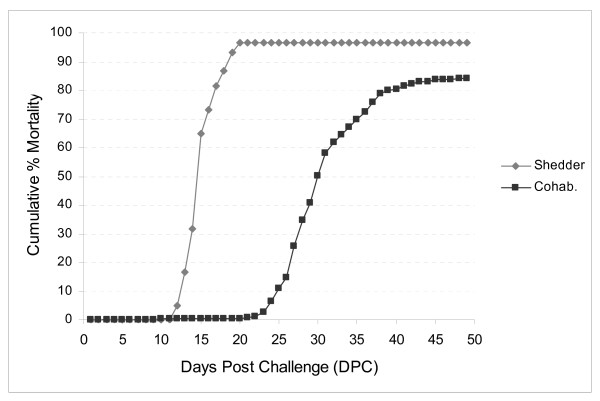
**Experimental ISAV challenge trial**. Mortality curves of the standardised ISAV cohabitation challenge among i.p.-injected carriers ('shedder', grey curve) and cohabitants ('cohab', black curve).

The cohabitant transmission of virus from fish injected with a previously tested viral load enabled the investigation of the immune response in fish after a natural route of infection. The chosen ISAV strain (Glesvaer 2/90) is well characterized and is classified among isolates inducing acute disease progression [4]. A cumulative mortality (CM) of 84% in the cohabitant fish confirms the highly pathogenic nature of this ISAV strain (Fig. 1). By termination 49 days post-infection (DPI) mortality rate had reached a plateau phase, but if continued mortality would likely have approached 100% (see virus load in section below). The injected fish started to die at 12 DPI and at 20 DPI the cumulative mortality had reached 97%. Cohabitants started to die at 21 DPI when nearly all (97%) carrier fish were dead. There was no mortality in the control tank. Tanks were monitored continuously and only moribund fish were sampled for RNA extraction. Based on experience from similar experiments, sampling was divided into three stages: early mortality (EM, 0–10% CM), intermediate (IM, 35–55% CM) and late mortality (LM, 75–84% CM).

### Virus load

**Figure 2:**
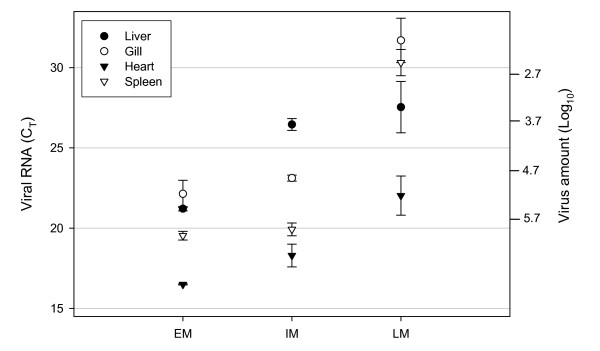
**Viral loads analysed by real-time qPCR**. Viral RNA levels of segment 6 in liver, gill, heart and spleen of four fish from EM, IM and LM stages. Levels are given as cycle threshold (C_T_) values (left Y-axis) and log_10 _number of viral particles (right Y-axis) based on a standard curve of C_T _against titrated *in vitro *infected (Glesvaer 2/90 isolate) ASK (Atlantic salmon kidney) cells.

The ISAV pathology in sampled fish was confirmed by clinical examination. Levels of infection in four random fish from the EM, IM and LM stages were determined by real-time qPCR analysis of viral RNA in all tissues (Fig. 2). This is a very sensitive and precise assay, reported to be 100-fold more sensitive than end-point RT-PCR [15] which has a threshold detection in the range of 0.01–0.1 TCID_50 _[16]. Low standard deviations of C_T _(cycle threshold) values within each stage indicated that viral RNA levels were similar between individuals and justified the comparison of gene expression between the three groups of mortalities. In general, there was a high viral replication in EM and IM stages with no significant difference between these groups, while a marked reduction was observed in LM (except for liver). Levels in survivor fish (16%, not moribund) were similar to LM (data not shown). When transforming C_T _values into viral particles based on a standard curve of C_T _from *in vitro *infected cells this corresponded to a 1000-fold reduction of viral particles in spleens only within 6 days (from 31–37 DPI), given that cohabitants were infected simultaneously. No significant difference was observed between levels of two ISAV segments (HA and NS1) (data not shown).

### Gene expression analyses

Microarray analyses were conducted on gill, heart, liver and spleen samples pooled from six individuals for each tissue, and on individual samples of liver (n = 6). The former indicated tissue-specificity of responses while the latter allowed for statistical assessment of differences between the early and late mortalities (EM and LM). Primary data was submitted to GEO and the processed results are available in Additional file 1. Genes with differential expression between EM and LM from microarray analyses were verified by real-time qPCR on individual heart samples (n = 4), and additional genes for adaptive immune responses not represented on the microarray were analysed on individual spleen samples (n = 6) from all stages (EM, IM and LM).

### Early mortalities show induced innate antiviral and stress responses

**Figure 3:**
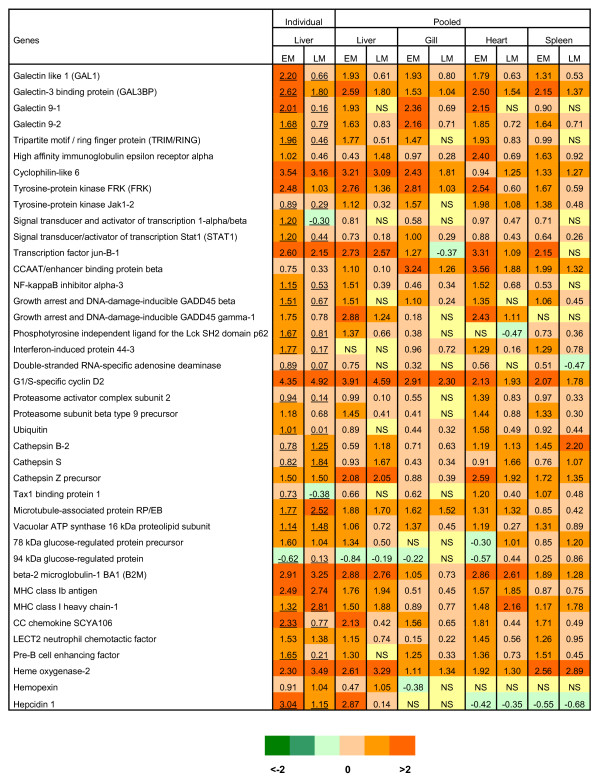
**EM-induced genes with similar expression changes across tissues**. Data are log-ER (Expression Ratio). Microarray analyses were conducted on individual liver samples (the group means are shown, n = 6; underlined values mean significantly different groups, p < 0.05) and in pooled samples (n = 6 per tissue) of liver, gill, heart and spleen (the average values of two slides are shown). Up- and down-regulated genes are highlighted with orange and green scales, as shown below the figure. NS means not significant (p < 0.01, t-test, 6 spot replicates per gene).

**Figure 4:**
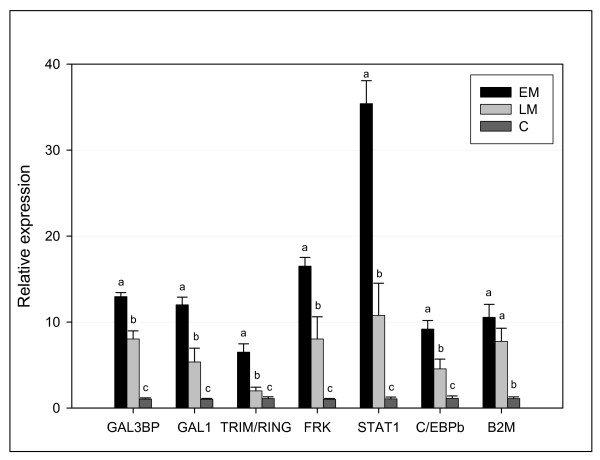
**Real-time qPCR analysis of early induced genes in heart from EM and LM**. Selected genes from microarray were; galectin-3 binding protein (GAL3BP), galectin 1-like (GAL1), tripartite motif protein/ring finger protein (TRIM/RING), tyrosine-protein kinase FRK (FRK), signal transducer/activator of transcription 1 (STAT1), CCAAT/enhancer binding protein beta (C/EBPb), beta-2 microglobulin-1 BA1 (B2M). Data are mean relative expression ratios ± SE of 4 infected individuals relative to 4 controls (C) per stage, normalized against 18S rRNA levels and adjusted for PCR efficiency. Bars with different letters are significantly different (p < 0.05).

Many genes were regulated across all tissues and a large fraction of these showed markedly higher expression levels in EM compared to LM (Fig. 3). Among these were several genes related to cellular stress (transcription factor jun-B, GADD45-β and -γ, 78 kDa and 94 kDa glucose-regulated proteins) as could be expected according to their involvement in pathological alterations. Up-regulation of several proteins involved in extracellular transport (Tax binding protein, microtubule-associated protein RP/EB, vacuolar ATP synthase and 78 kDa glucose-regulated protein) was consistent with reticular stress as observed in various viral diseases [17]. Given the high viral replication and mortality in EM it was noteworthy how many genes with pivotal roles in innate antiviral responses were highly induced at this stage without conferring any protection (Fig. 3, 4). The src-type tyrosine kinase Jak and signal transducer/activator of transcription Stat1 play a key part in induction of IFN-dependent genes [18,19]. Up-regulation of interferon induced protein 44 and double-stranded RNA-adenosine deaminase (ADAR-1), one of the key antiviral effectors, was additional evidence for activation of the IFN-axis. Similar profiles were seen in src-related tyrosine-protein kinase FRK and ligand for Lck SH2 domain p62 which regulate various immune responses, being involved in a complex network of signal transduction pathways. Further downstream, up-regulation of several IFN-responsive genes involved in antigen processing and presentation (β2 m encoding MHCI light chain, tapasin, proteasome activator complex, ubiquitin, cathepsins) indicated an early activation of adaptive immunity in EM. Broad induction of GADD45-β and -γ could contribute to increased survival of immune cells through interaction with NF-κB pathway [20,21]. Further evidence for early immune cell activation was increased leukocyte migration and recruitment (up-regulation of chemokines and receptors; SCYA110-2/CCL7-like, SCYA106/CCL21-like, CXCR4-like) and monocyte and lymphocyte maturation and activation (up-regulation of regulator of G-protein signalling/RGS1, Fc-gamma receptor/CD64, TNFR5/CD40, LECT2). A number of EM-induced genes could be involved in other functions along with antiviral defense; galectins are small, highly conserved proteins that bind sugars and other ligands and can work as both positive and negative regulators of various immune functions [22,23]. Coordinated regulation of galectins and interferons was reported in mammals [24,25].

### Late survival is associated with reduced viral load and activation of adaptive immunity

**Figure 5:**
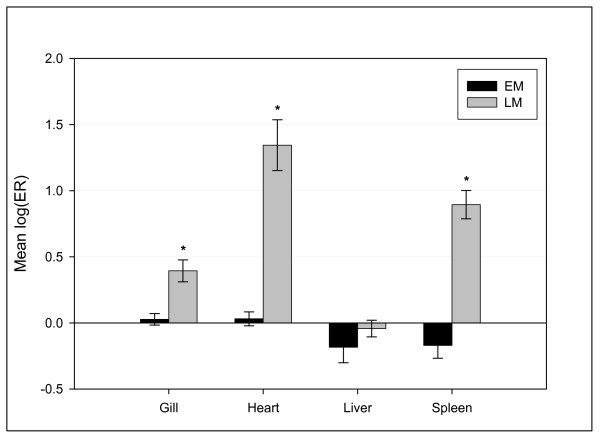
**Immunoglobulin-related transcripts induced in LM**. Microarray expression (mean log(ER) ± SE) in gill, heart, liver and spleen (6 individuals pooled per tissue) of 11 immunoglobulin-related clones designated by the Unigene clusters and most similar mammalian proteins: Omy.9391 (similar to Ig-kappa chain V-III region VG and Ig-kappa chain C region), Omy.15640 (similar to Ig-gamma-2 chain C region and Ig-mu chain C region membrane-bound), Ssa.78 (similar to Ig heavy chain V-III region HIL), Ssa.709 (similar to Ig-kappa chain V-I region WEA and to Ig-kappa chain V-IV region B17-2), Omy.416 (similar to Ig-kappa chain V-IV region JI), Omy.23312 (similar to Ig-kappa chain V-IV region B17-1), Omy.30091 (similar to Ig-kappa chain V-IV region Len) and Omy.11287 (similar to Ig-mu heavy chain disease protein). Asteriks indicates significantly different groups (p < 0.0001).

A group of genes induced at the LM versus EM stage was of major interest since they could point to responses important for resistance or late survival. Microarray analyses of heart, spleen and gills revealed a coordinated induction of Ig-related genes (Fig. 5). A gene similar to CD179β (Ig-kappa chain V-I; see Additional file 1) encodes a receptor found on the surface of pre- and pro-B cells involved in signal transduction and differentiation, allelic exclusion at the Ig heavy chain locus, and promotion of Ig light chain gene rearrangements [26]. Functionally related were also two other similarly induced transcripts of the IgM heavy chain (Ig-mu heavy chain disease protein and Ig-mu chain C region membrane-bound). The CD84 leukocyte antigen is also interesting in this context, being highly expressed in certain B-cell subsets and belonging to the recently discovered family of signaling lymphocyte activating molecule (SLAM)-related receptors (SRR), a group of surface molecules whose main function seems to be the fine-tuning of lymphocyte responses [27]. Among this class of LM induced genes were also five Ig-kappa genes involved in B-cell receptor (CD79α/β) signalling (Ig-kappa chains V-IV region B17-1+2, V-III region VG, V-IV region JI and V-IV region Len). Collectively, the expression profiles of these genes indicated that a general activation of B-lymphocyte maturation and humoral immunity occurred among the late survivors. However, the gene homologous to the B-cell receptor itself was unregulated or down-regulated in spleen together with some B-cell regulatory genes. Notably, MHC class II (α- and invariant chain Il), which is the mediator of Ag-presentation in B-cells (and other APCs), was also down-regulated in spleen at both EM and LM stages, but was induced in heart of LM but not EM.

**Figure 6:**
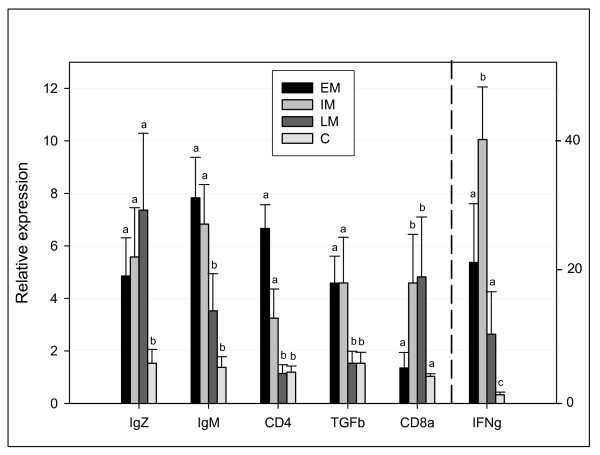
**Real-time qPCR analyses of genes related to adaptive immunity from spleen at all stages**. Selected genes were; heavy chains of immunoglobulin zeta (IgZ) and immunoglobulin mju (IgM), T cell coreceptors CD4 (CD4) and CD8alpha (CD8a), T_H_2 cytokine transforming growth factor beta (TGFb), T_H_1 cytokine interferon gamma (IFNg). Data are mean relative expression ratios ± SE of 6 infected individuals relative to 6 controls (C) per stage, normalized against 18S rRNA levels and adjusted for PCR efficiency. IFN-γ expression is shown with own Y-axis for easier comparison between genes. Bars with different letters are significantly different (p < 0.05).

To further detail the features of the adaptive immune responses observed in LM we designed qPCR primers for well-known genes of humoral (IgM, -Z and -D heavy chain) and T-cell mediated (CD8^+ ^CTL response; CD8α, CD4^+ ^T_H_1 response; IFN-γ and T_H_2 response; TGF-β) immunity. For this analysis we also included fish in the IM stage and increased the number of individuals (6 per stage) to see if the viral clearance observed in LM was explained by a general activation of adaptive immunity in IM. The results showed that IgM and IgZ, but not IgD (data not shown), were induced at all stages versus control but in opposite manners (Fig. 6). IgM mRNA peaked at EM and declined towards the later stages (IM and LM) while IgZ was steadily induced towards peak levels at LM. Kinetics of IgM expression was similar to CD4 and TGF-β. In contrast, expression of CD8α was markedly induced from EM to IM/LM stages and correlated with IFN-γ expression (from the EM to IM stage). It must be noted that the significance of the data varied due to individual variation, but all qPCRs were replicated three times in both the reverse transcription step and in PCR, giving identical results. Thus, the pronounced reduction in virus load from IM to LM might be explained by both developing antibody responses (IgZ) and activation of T-cell mediated immunity at IM, either through a specific CD8^+ ^cytotoxic and/or T_H_1 response. Notably, this activation corresponded with induced antigen presentation, e.g. a broad elevation of MHCI mRNA levels across all tissues. However, MHCII (α- and invariant chain) was generally down-regulated in late survivors except for up-regulation in heart LM, the tissue with highest viral RNA levels.

### Late mortalities show reduced hepatic expression of chaperones and genes involved in metabolism of steroids, lipids and xenobiotics

A suite of chaperones from different groups (heat shock proteins and cognates, DnaJ homologs and T-complex proteins) showed consistently lower expression levels in liver from LM compared to EM (Table 1). Chaperones are essential for the life cycle of viruses, being involved in replication, translation, transport and assembly of viral proteins. Viruses from diverse groups are known to use chaperones of the hosts and/or produce own proteins with protein folding properties [28-31]. Chaperones are commonly used as markers of cellular stress. Although fish from all study groups was sampled in a moribund state, higher expression levels of chaperones suggested more severe stress in individuals with early mortality.

**Table 1:** Microarray expression of genes encoding chaperones in liver. The data are mean log(ER) ± SE.

Genes	EM	LM	LM-EM^1^
Heat shock protein HSP 90-beta	0,97 ± 0,22	-0,15 ± 0,13	-1,13
Heat shock cognate 70 kDa	0,35 ± 0,19	-0,70 ± 0,07	-1,05
Heat shock cognate 71 kDa	0,42 ± 0,21	-0,60 ± 0,10	-1,02
Heat shock 70 kDa-8	0,01 ± 0,15	-0,89 ± 0,09	-0,90
Heat shock protein 75 kDa-2	-0,87 ± 0,08	-1,36 ± 0,10	-0,48
Heat shock factor 2-2	-0,78 ± 0,10	-1,28 ± 0,04	-0,50
DnaJ homolog subfamily B member 11	0,98 ± 0,25	-0,02 ± 0,27	-1,00
DnaJ homolog subfamily C member 9	-1,02 ± 0,16	-1,69 ± 0,13	-0,68
DnaJ homolog subfamily A member 2	0,24 ± 0,11	-0,40 ± 0,09	-0,64
T-complex protein 1, subunit 5	0,86 ± 0,12	0,27 ± 0,12	-0,59
T-complex protein 1, gamma subunit	-0,53 ± 0,12	-1,02 ± 0,10	-0,49

^1^Difference of mean log(ER) between two groups. All mean log(ER) were significantly lower in LM (p < 0.001, t-test, n = 12).

In liver ISAV also affected genes involved in steroid and lipid metabolism (Table 2). Functional consequences of the potential decrease in metabolism of highly unsaturated fatty acids (delta-6 fatty acid desaturase), bile (sodium/bile acid cotransporter) and steroids (3-oxo-5-beta-steroid 4-dehydrogenase and estradiol 17 beta-dehydrogenase) are unclear given the complex interaction of these pathways with virus replication, immunity and stress. However since these genes showed little or no difference between EM and LM, their expression apparently did not influence resistance to virus in our experiment. Disparity between fish with different survival times was better seen in a group of genes associated with metabolism of eicosanoids, which may play both positive and negative roles in viral diseases as regulators of pathogen life cycle (replication) and inflammation [32,33]. Cytochromes P450 can have different modes of action being involved in metabolism of eicosanoids and xenobiotics as well carboxylesterase HU1 [34]. C/EBP delta, an agonist of peroxisome proliferator-activated receptor is one of the key factors required for differentiation of adipocytes [35]. This gene was suppressed in contrast to alpha/beta type (Fig. 3, 4). Differential regulation of C/EBP isoforms has been reported under various acute conditions [36,37].

**Table 2:** Hepatic microarray expression of genes implicated in metabolism of lipids and steroids. The data are mean log(ER) ± SE.

Genes	EM	LM	LM-EM
CCAAT/enhancer binding protein delta^1^	-0,26 ± 0,19	-1,07 ± 0,20	-0.81
Leukotriene B4 receptor^1,2^	-1,17 ± 0,11	-1,85 ± 0,16	-0.68
Carboxylesterase HU1a^1^	-0,48 ± 0,11	-1,42 ± 0,37	-0.94
Delta-6 fatty acid desaturase	-1,16 ± 0,11	-1,04 ± 0,19	0.12
Cytochrome P450 2K4^1,2^	-0,36 ± 0,15	-1,19 ± 0,16	-0.83
Cytochrome P450 2F1^1,2^	-1,09 ± 0,13	-1,85 ± 0,19	-0.76
3-oxo-5-beta-steroid 4-dehydrogenase	-1,62 ± 0,43	-2,07 ± 0,50	-0.45
Estradiol 17 beta-dehydrogenase	-0,71 ± 0,13	-0,93 ± 0,21	-0.22
Prostaglandine D synthase	-1,09 ± 0,23	-0,92 ± 0,27	0.17
Progesterone receptor component 2^1^	-0,67 ± 0,16	-1,15 ± 0,17	-0.48
Estrogen-responsive B box protein^1^	-0,45 ± 0,10	-1,11 ± 0,15	-0.66
D-3-phosphoglycerate dehydrogenase^1^	-0,86 ± 0,12	-1,23 ± 0,06	-0.37
Sodium/bile acid cotransporter^1^	-0,94 ± 0,07	-1,30 ± 0,08	-0.36
All-trans-13,14-dihydroretinol saturase^2^	-0,98 ± 0,16	-1,27 ± 0,09	-0.29
5-lipoxygenase activating protein^1,2^	0,34 ± 0,14	1,55 ± 0,32	1.21
Annexin A1^1,2^	-0,16 ± 0,09	1,59 ± 0,55	1.75

^1^Significant difference between groups (p < 0.05, t-test, n = 12). ^2^Involved in metabolism of eicosanoids

### Development of prognostic markers

**Figure 7:**
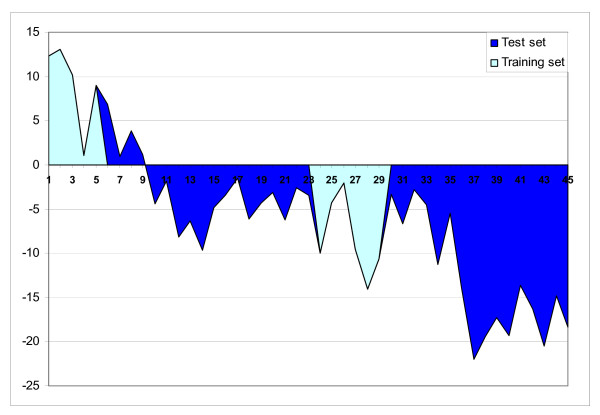
**Class prediction based on qPCR analyses of eight genes in liver**. The training and test sets were analyzed in two independent experiments indicated with different colours. The predictor function was constructed by linear discriminant analysis of the training group and verified using the test group. Numbers of individuals: 1–9 – EM, 10–23 – IM, 24–35 – LM and 36–45 – control.

Microarray screening with subsequent qPCR analyses of candidate genes is a widely used approach for developing prognostic markers. We wished to try a hypothesis-free strategy meaning that gene expression profiles were used with no surmise on gene functions. Therefore the candidate genes (Table 3) were chosen exclusively by their expression profiles (differences between EM and LM). Six individual liver samples from each of EM and LM stages were used as a training set in a linear discriminant analysis. For this analysis a function was constructed which took positive values in EM and negative values in LM while intercept with X-axis marked a boundary between these groups (Fig. 7). Furthermore, the classifier was verified in an independent test set, which included fish from all stages and controls. The uninfected fish were well separated from the challenged, and as expected, the values of predictor function were negative as well as in LM. The individuals from EM and LM were all assigned to the correct classes. Fish with early mortality formed a sharply outlined group however IM was not separated from LM. The genes were ranked by Wilks' coefficients. To evaluate the minimum number of genes required for the correct class assignment we used forward and reverse procedures. The genes were either added or deleted one by one starting with respectively highest and lowest ranks, the predictor function was re-calculated and the results were compared to those shown in Fig. 7. In both cases, four genes (5-lipoxygenase activating protein, cytochrome P450 2K4, galectin-9 and annexin A1) were determined as the minimum number required to build the function that ensured robust class prediction. These genes changed expression in different directions (Table 2) and interestingly, three of four genes were related to metabolism of eicosanoids which are inflammatory regulators of lipid origin.

**Table 3:** Real-time qPCR primers used in the study

**Genes**	**Primers**	**GenBank**
ISAV segment 6 ^a^	5-AGGCCAAAAACGGAAATGGA-3	AF220607
	5-CCGTCAGTGCAGTCATTGGTT-3	
18S rRNA ^b^	5-GCCCTATCAACTTTCGATGGTAC-3	AJ427629
	5-TTTGGATGTGGTAGCCGTTTCTC-3	
Galectin-3 binding prot ^c^	5-CCAGACCAACAGTGTTCACTTCAGC-3	BX079375
	5-ACGTGAAAGACATACCTGCCCTCAC-3	
Galectin-like 1 ^c^	5-CAGCAACCCTTCTTCAATCCGAGA-3	CA344100
	5-TCTCCCTGTCACAGTGATGGTCTTC-3	
Tripartite motif/ring finger protein ^c^	5-TGTTCTGTTGCTCCGTCTGTCTGGA-3	CA376536
	5-TTCAGCCAGCACGGTGTTTCTCTTC-3	
Tyr-protein kinase FRK ^c^	5-TAGACATGGCACCATGGACCCTC-3	CA349577
	5-GGGTTCTTCAGTGCAGACAGCCA-3	
STAT1 ^c^	5-GAACATGGAGGAGTCCAATGGAAGC-3	CA343225
	5-GGACCCTCATTTGATCTGTTGCCT-3	
CCAAT/enhancer binding protein beta ^c^	5-TACGTCCTGGGCTATCCTGAACTGC-3	CA348284
	5-CCAGACGAACCGTTGTTGTCCA-3	
Beta-2-microglobulin ^c^	5-TCGTTGTACTTGTGCTCATTTACAGC-3	AF180478
	5-CAGGGTATTCTTATCTCCAAAGTTGC-3	
IgM heavy chain (CH) ^d^	5-ATACGGTGACCCTGACTTGCTACGT-3	S48652
	5-TTCTCTCCACCGGCTCATCATCA-3	
IgZ CH-like ^d^	5-AGCACCAGGGACAAACCACCAT-3	CA372094
	5-TTCACACTCGGTGGGTTCAGAGTC-3	
IgD CH ^d^	5-TCTTCAGGAGCTGAGGACAGATGGA-3	AF278717
	5-AAACCCATCCACCTTCCAGCTGA-3	
CD4 ^d^	5-TGCATTGTTCCTCTCTTCCACAGC-3	AY973028
	5-CCGTCCCAAGGTACCATAGTACCAA-3	
TGF-beta ^d^	5-AATCGGAGAGTTGCTGTGTGCGA-3	EU082211+
	5-GGGTTGTGGTGCTTATACAGAGCCA-3	AJ007836
CD8-alpha ^d^	5-CGTCTACAGCTGTGCATCAATCAA-3	AY693391
	5-GGCTGTGGTCATTGGTGTAGTC-3	
Interferon-gamma ^d^	5-TTCAGGAGACCCAGAAACACTAC-3	AJ841811
	5-TAATGAACTCGGACAGAGCCTTC-3	
GRB2-related adaptor protein 2 ^e^	5-TGACTTTACTGCCACTGCTGAGGAC-3	CA353121
	5-CAGTCATCATTGGTGCCCAAGATC-3	
Annexin A1-1 ^e^	5-CTCCAGGAAATTGAACACCGCGA-3	CA364941
	5-AAGGCTGCGATGAAGGACATGGT-3	
5-lipoxygenase activating protein ^e^	5-TCTGAGTCATGCTGTCCGTAGTGGT-3	CA369467
	5-CCTCCCTCTCTACCTTCGTTGCAAA-3	
Regulator of G-protein signaling 1-1 ^e^	5-GACTCCTAACCTCCAATGCTTCGAC-3	CA383094
	5-CGAATCTCTCTCCATCAGCCCATA-3	
Cytochrome P450 2K4-1 ^e^	5-TTCACCCTCCACCCTTCACCTC-3	EV384586
	5-ATCTCAGACCCGGCTCACAGCA-3	
Galectin-9 ^e^	5-TCGCTGATTGTGAATGGTGCTCAC-3	CX035552
	5-CAGGGTTGGAGAAGGCAATGGATT-3	
Heat shock protein HSP 90-beta-2^e^	5-GAACCTCTGCAAGCTCATGAAGGA-3	BX074486
	5-ACCAGCCTGTTTGACACAGTCACCT-3	
Cathepsin S ^e^	5-CGAAGGGAGGTCTGGGAGAGGAAT-3	CA355014
	5-GCCCAGGTCATAGGTGTGCATGTC-3	

^a ^Viral RNA quantification. ^b ^Reference gene. ^c ^Microarray confirmation and EM-induced genes, Fig. 4. ^d ^Adaptive immune responses all stages, Fig. 6. ^e ^Development of class prediction, Fig. 7.

## Discussion

We searched for the host factors that may account for different susceptibility and resistance to ISAV in Atlantic salmon. This question is of great importance for the development of protective strategies in aquaculture. With the exception of one study that reported possible association of specific MHC class I alleles with survival [7] there is no evidence in the literature of defensive/offensive responses that could underlie individual variation resistance to ISAV. An advantage of research on infectious diseases in fish is the possibility to challenge large numbers of individuals and to locate groups with markedly different performance. Survival time is commonly used as a simple measure of resistance based on the assumption that it is determined principally by antiviral defense and/or severity of the pathological alterations. One cannot neglect the possibility that differences in survival times are largely stochastic by nature. Then given similar physiological conditions (all fish were sampled in a moribund state) we could expect comparable phenotypes in the groups with different survival times; however our studies revealed marked differences between the study groups (stages). Survival time can depend on the level of infection determined by the probability and time-point of encounter with pathogens and/or with active or passive protective mechanisms. Notably, an identical challenge test as used here was previously shown to be very efficient in that all cohabitants were simultaneously infected [38], justifying that survival times could be explained by protective mechanisms. The viral loads were remarkably lower in LM however we did not find significant difference between EM and IM (except for liver). Therefore viral load was an important, but not the only factor, influencing survival times. Dramatic activation of the innate immune responses in EM fish was a notable and intriguing finding. Such responses were observed at different cellular levels with possible affects on the binding of ligands, perception and transduction of signals, regulation of gene expression and antiviral effectors. Importantly, many genes with significantly higher expression levels in EM are known to be IFN-dependent and as expected these responses were characterised by low tissue specificity. Our result was in line with a recent finding that induction of type I IFN and IFN-dependent genes in ISAV infected fish and cells did not provide protection against virus [8,39]. In addition, knowledge is emerging on potential IFN-antagonistic mechanisms of ISAV's NS1 protein [9], which is well-known from influenza [40]. We do not know whether strong antiviral responses were detrimental to fish. Such a possibility cannot be excluded, however, it is likely that activity of the IFN axis was directly related to the viral load (which was greater in EM). Induction of galectins was a striking feature of the early broad tissue responses to ISAV. Regulation of these genes with IFN was reported in higher vertebrates [24,25] and recently in trout macrophages [41]. It is known that galectins can be used by virus [42]. However, strong responses in phylogenetically remote species (fish and mammals) to diverse viruses such as ISAV in salmon and rhabdovirus (VHSV) in trout [43] make such a possibility very unlikely. Proteins with ability to bind diverse ligands can work as factors that restrict the propagation of the virus. Studies on these mechanisms, which unlike the generalized antiviral responses appear more pathogen-specific, represent a rapidly emerging area. Probably the best known achievement is identification of tripartite motif proteins and cyclophilin A, that may account for inter-specific differences in resistance to HIV [12]. Interestingly, we observed up-regulation of ring finger/tripartite motif protein and cyclophilins, but without association to increased survival. We cannot reject the potential variation in the ability to restrict propagation of ISAV. To assess this possibility it will be necessary to screen for viral loads in a large number of challenged individuals, preferably from families with markedly different resistance. Overall, it was obvious that the innate antiviral responses did not provide protection to ISAV-infected salmon.

Apart from active protection, survival time may depend on the tolerance or ability to endure high viral loads, and comparison of the hepatic responses tended to support this possibility. Regulation of heat shock proteins plus other chaperones regarded as evidence for cellular stress, is a common feature of various viral diseases [44]. These chaperones changed expression in both directions, however levels in LM were consistently lower than in EM. We could also see evidence for less severe inflammatory responses in LM. While many inflammatory regulators showed similar profiles at all stages, there was a difference in expression of genes involved in the metabolism of eicosanoids, which have been found to play an important part in pathogenesis of many viral diseases [33]. Importantly, two genes induced in LM (5-lipoxygenase activating protein and annexin A1) were identified as the most valuable markers for the discrimination of groups with different survival times. Both genes encode for proteins associated with phospholipase A2 and are regarded as important targets for anti-inflammatory therapy [45,46]. Annexin AI inhibits phospholipase A2, which releases arachidonic acid from cellular membranes. Arachidonic acid is subsequently transformed to prostaglandins, thromboxane and leukotrienes collectively termed eicosanoids (reviewed in [47]) and 5-lipoxygenase activating protein plays a key part in one of these pathways. Differences between EM and LM were seen in several more genes of eicosanoid metabolism while prostaglandin D synthase was equally down-regulated in both groups.

Microarray analyses revealed only one group of genes (immunoglobulin-related) that were higher induced in LM compared to EM. Changes in levels of these transcripts could be due to maturation and/or migration of immune cells to the heart and spleen, the two most heavily infected organs, and suggested an activation of adaptive immune responses in late survivors. The microarray includes eleven Ig clones corresponding to seven Unigene clusters. The exact roles of these salmon genes are not known, however their putative mammalian homologs are involved in early B-cell development. Apart from mediating antibody responses, mature B cells are crucial for activation of effector helper T cells [48]. Equally important was the fact that induction of these Igs correlated with a viral clearance among LM fish. This prompted an extended qPCR analysis including all arms of adaptive cellular immunity, and showed that the expression kinetics of IgM, -D and -Z heavy chains, the most likely mediators of antibody responses in salmon, were quite different. The reason for this is at best speculative, but it could indicate different roles for IgM and IgZ in salmonid immunity (e.g. innate natural versus humoral neutralizing antibodies). Perhaps more interesting was the induction of CD8α and IFN-γ, but not TGF-β (T_H_2 marker), in IM and LM fish which correlated with viral clearance in LM fish. An optimal T_H_1 response, consisting of virus-specific (IFN-γ)-secreting CD4^+ ^T cells and cytotoxic CD8^+ ^T cells that lyse virus-infected cells [49], is crucial for clearance of influenza virus infection in humans [50]. Further strengthening the significance of a cytotoxic response was the fact that MHC class I, the key activator of CD8^+ ^T cells, was consistently induced in all tissues and stages. These results also support recent observations that ISAV survival correlated more strongly to the level of cell-mediated responses than did humoral responses [4].

Collectively, these findings demonstrate the power of functional genomics in untangling the complexities of virus-host interactions and viral pathogenesis. From analysis of gene expression profiles we suggest that common features of highly pathogenic ISAV include high viral replication, dramatic induction of innate immunity without protection and subsequent activation of cell-mediated immunity and reduced inflammation associated with viral clearance. Similarities to influenza pathogenesis are striking; fatalities of the 1918 influenza virus is caused by rapid replication kinetics resulting in an excessively vigorous innate immune and inflammatory response that contributes to severe tissue damage, disease and death [51,52].

The need for prognostic and diagnostic tools for control of aquaculture diseases is urgent. We compared consequences of the acute and protracted forms of ISAV infection, but to search for prognostic markers our experimental design was not appropriate as analyses should have been conducted before challenge. Nonetheless, we regard this study as a step towards this task, which can help to clarify a number of important questions. An ideal strategy for prediction of resistance would be an in vitro test, such as stimulation of primary cultures (e.g. peripheral blood cells) with ISAV and subsequent gene expression analysis. However because we did not find any broad-tissue responses associated with improved resistance the feasibility of such approach is ambiguous. The LM group was characterized by activation of adaptive immune responses which are systemic by nature and require complex interactions of different cellular elements and humoral factors. Given the pivotal role of acquired responses, the development of animal models will be essential for studies on fish resistance to viral pathogens. We used materials from this experiment as a pilot study for the development of predictors based on microarray and qPCR analyses. Four genes were sufficient for accurate class assignment and importantly, this was achieved using linear discriminate analyses, a gold standard approach to tasks of this kind.

## Conclusion

In conclusion, three groups with different survival times were characterized by distinct phenotypes suggesting that resistance at early and late stages of ISAV could be explained by different molecular host determinants. Early mortality associated with highly pathogenic form of ISAV was characterized by high viral replication, dramatic up-regulation of innate immune mechanisms and cellular stress. Intermediate mortalities shared common features with EM (high viral load) in contrast to the late group of survivors where a significant viral clearance coincided with activation of adaptive cellular immunity and reduced inflammation. Overall, development of adaptive immune responses in salmonid fish requires several weeks after pathogen challenge, which is consistent with our results. Ability to survive to this point is likely to have crucial importance for the outcome of disease.

## Methods

### Experimental infection

Tissue samples from control and ISAV infected fish originated from a reference challenge trial performed at VESO Vikan (Veterinary Science Opportunities, Namsos, Norway). The trial was approved by The National Animal Research Authority (see Availability and requirements section for URL) according to the 'European Convention for the Protection of Vertebrate Animals used for Experimental and other Scientific Purposes' (EST 123). Unvaccinated juvenile Atlantic salmon (average size 22.8 gram at start) were kept in separate tanks at 12°C under controlled conditions (water flow, fish density etc.) and acclimatised for one week before challenged. In one tank, 300 fish (hereafter referred to as cohabitants) were cohabitated with 60 marked fish each i.p.-injected with a virus dose of 7.2 × 10^3 ^TCID_50 _of a third passage of ISAV strain Glesvaer 2/90 (National Veterinary Institute, Oslo, Norway). A separate tank contained 40 unvaccinated control fish of same size and origin which were kept under identical conditions (density, temperature, water flow and feeding) as challenged fish. Standardized tissue sampling (heart, spleen, gills and liver) was performed from 12, 24 and 12 moribund fish from respectively EM (0–10% CM), IM (35–55% CM) and LM (75–84% CM) stages (cohabitants) and similarly from half the number of controls at the same time-points as challenged fish. Samples were immediately stored in >10× excess volume of RNAlater (Invitrogen, Carlsbad, CA, USA) at 4°C overnight following -20°C until RNA extraction. All fish were negatively tested for immune status by ELISA testing for *Vibrio salmonicidae*, *Vibrio anguillarum *O1 and O2, *Moritella viscosa *and *IPNV *before challenge. Moribund fish were confirmed clinically positive for ISAV. No mortality was observed among control fish.

### RNA extraction and cDNA synthesis

Total RNA from tissue was isolated using PureLink Micro-Midi kit (Invitrogen) using manufacturer's protocols and guidelines. RNA quantity was measured by Nanodrop (Thermo Fisher Scientific, Waltham, MA, USA) and integrity confirmed by gel electrophoresis. Samples were precipitated and stored under ethanol at -70°C. cDNA synthesis was performed on 2 μg DNAse-treated (Turbo DNA-*free*™, Ambion, Austin, TX, USA) total RNA using TaqMan^® ^Reverse Transcription reagents (Applied Biosystems, Foster City, CA, USA) and random hexamer primers, according to manufacturer's protocol.

### Microarray analyses

The salmonid fish cDNA microarray SFA2.0 immunochip contains 1800 unique clones printed each in six spot replicates. The genes were selected by their functional roles and the platform is enriched in a number of functional classes such as immune response (236 genes), cell communication (291 genes), signal transduction (245 genes) and receptor activity (126 genes), apoptosis (120 genes), cell cycle (76 genes), protein catabolism (90 genes) and folding (70 genes) and response to oxidative stress (39 genes). The gene composition and sequences are provided in GEO (GPL6154). Microarray analyses were conducted on fish from EM and LM stages. A common reference design of hybridization was applied and RNA pooled from 6 uninfected fish per tissue and per stage was used as control. Pooled samples of gill, liver, heart and spleen (equal amounts of RNA from 6 individuals per tissue) were analyzed in a dye-swap design; two slides were used for each sample with reverse assignment of dyes. Individual comparisons (6 fish from EM and 6 fish from LM) were done on liver samples in a single-slide format and one slide per each sample was used. The samples (20 μg RNA in each) were labeled with Cy3-dUTP and Cy5-dUTP (Amersham Pharmacia, Little Chalfont, UK) using the SuperScript™ Direct cDNA Labeling System (Invitrogen). The cDNA synthesis was performed at 43°C for 3 hours in a 20 μl reaction volume, followed with RNA degradation with 0.2 M NaOH at 37°C for 15 min and alkaline neutralization with 0.6 M Hepes. Labeled cDNA was purified with Microcon YM30 (Millipore, Beford, MA, USA). The slides were pretreated with 1% BSA fraction V, 5 × SSC, 0.1% SDS for 30 min at 50°C and washed with 2 × SSC for 3 min and 0.2 × SSC for 3 min at room temperature and hybridized overnight at 60°C in a cocktail containing 1.3 × Denhardt's, 3 × SSC, 0.3% SDS, 0.67 μg μl^-1 ^polyadenylate and 1.4 μg μl^-1 ^yeast tRNA. After hybridization slides were washed at room temperature in 0.5 × SSC and 0.1% SDS for 15 min, 0.5 × SSC and 0.01% SDS for 15 min, and twice in 0.06 × SSC for 2 and 1 min, respectively. Scanning was performed with GSI Lumonics ScanArray 4000 (PerkinElmer Life Sciences, Zaventem, Belgium) and images were processed with GenePix Pro 6.0 (Axon, Union City, CA, USA). The spots were filtered by criterion (*I*-*B*)/(*S*_*I*_+*S*_*B*_) ≥ *0.6*, where *I *and *B *are the mean signal and background intensities and *S*_*I*_, *S*_*B *_are the standard deviations. The low quality spots were excluded from analysis and genes presented with less than three high quality spots on a slide were discarded. After subtraction of median background from median signal intensities, the expression ratios (ER) were calculated. Lowess normalization was performed first for the whole slide and next for twelve rows and four columns per slide. The differential expression was assessed by difference of the mean log-ER from zero (6 spot replicates per each gene, Student's t-test, p < 0.01). The log-ER values in individual liver samples were compared with t-test (p < 0.05). Data were submitted to GEO (GPL6154).

### Quantitative real-time RT-PCR (qPCR)

qPCR primers (Table 3) were designed using the Vector NTI software (Invitrogen) and synthesized by Invitrogen. To avoid genomic DNA amplification, amplicons were when possible placed over introns and product size and specificity were confirmed by agarose gel electrophoresis and melting curve analysis (Tm calling; LightCycler 480, Roche Diagnostics, Mannheim, Germany). PCR efficiency (E) was determined from tenfold serial dilutions of cDNA for each primer pair and calculated according to Rasmussen [53]. Each pair of primers was tested on different samples in the same plate to ensure optimal reproducibility and repeated testing was performed on whole template setup or a random selection of samples to ensure inter-assay reproducibility. As common reference gene 18S rRNA was used, which was previously optimised and validated [54]. Four potential reference genes were tested (18S rRNA, EF-1alpha and two other non-regulated genes from the microarray results; SEC13-related protein and NADH-ubiquinone oxidoreductase 19 kDa subunit) using the BestKeeper [55] and GeNorm [56] software, however only 18S rRNA met qualifications of stability. PCR assays were optimized using 2 × SYBR^® ^Green Master Mix (Roche Diagnostics) and varying amounts of cDNA and primer concentrations. Optimal PCR conditions in a 12 μl reaction volume were 3 μl 1:10 diluted cDNA for all assays (1:2000 for 18S rRNA). Primer concentrations were 0.4–0.6 μM. PCR was performed in duplicates (triplicates for E-curves) in 96-well optical plates on LightCycler 480 (Roche Diagnostics). Running conditions were 5–10 min pre-incubation following 40 cycles of 95°C for 5 sec, 60°C for 15 sec, 72°C for 15 sec. Cycle threshold (C_T_) values were calculated using both the fit points and second derivative methods (Roche Diagnostics), with respective rejection of C_T _values above 37 and 35. Relative expression of mRNA was calculated using the ΔΔC_T _method adjusted for E. Statistics was calculated using Unistat version 5.5 (Unistat, London, UK). Difference between groups was analyzed with ANOVA with subsequent Newman-Keuls test (p < 0.05). The design of analyses and numbers of samples are indicated in Table 3.

### Development and verification of predictors based on qPCR analyses

Eight genes were chosen for the development of predictors based on results from microarray analyses of the liver (Table 3). A training group included six fish from EM plus six fish from LM. The C_T _values as determined by qPCR were processed with linear discriminant analyses using Statistica 6.0. The prediction function was verified with independent samples from all study groups (10 individuals from control, 9 from EM, 14 from IM and 12 from LM).

## Availability and requirements

The National Animal Research Authority: 

## Competing interests

The author(s) declare that they have no competing interests.

## Authors' contributions

SMJ and AK designed the study, conducted gene expression analyses and drafted the manuscript. SA developed software for processing of microarray data and performed the statistical analyses. All authors read and approved the final manuscript.

## Supplementary Material

Additional file 1 complete results of microarray analyses. log-expression ratios (ER) and respective p-values Click here for file

## References

[B1] Kibenge FS, Munir K, Kibenge MJ, Joseph T, Moneke E (2004). Infectious salmon anemia virus: causative agent, pathogenesis and immunity. Anim Health Res Rev.

[B2] Rimstad E, Mjaaland S (2002). Infectious salmon anaemia virus. Apmis.

[B3] Kibenge FS, Kibenge MJ, McKenna PK, Stothard P, Marshall R, Cusack RR, McGeachy S (2001). Antigenic variation among isolates of infectious salmon anaemia virus correlates with genetic variation of the viral haemagglutinin gene. J Gen Virol.

[B4] Mjaaland S, Markussen T, Sindre H, Kjoglum S, Dannevig BH, Larsen S, Grimholt U (2005). Susceptibility and immune responses following experimental infection of MHC compatible Atlantic salmon (*Salmo salar L*.) with different infectious salmon anaemia virus isolates. Arch Virol.

[B5] Falk K, Dale OB Lack of receptor-destroying enzymatic  activity of ISA-virus is an important virulence factor for development of  ISA in Atlantic salmon. In Frisk Fisk Conference, Tromsø:  Jan 23-25th 2007; Norway.

[B6] Kristiansen M, Froystad MK, Rishovd AL, Gjoen T (2002). Characterization of the receptor-destroying enzyme activity from infectious salmon anaemia virus. J Gen Virol.

[B7] Grimholt U, Larsen S, Nordmo R, Midtlyng P, Kjoeglum S, Storset A, Saebo S, Stet RJ (2003). MHC polymorphism and disease resistance in Atlantic salmon (*Salmo salar*); facing pathogens with single expressed major histocompatibility class I and class II loci. Immunogenetics.

[B8] Kileng O, Brundtland MI, Robertsen B (2007). Infectious salmon anemia virus is a powerful inducer of key genes of the type I interferon system of Atlantic salmon, but is not inhibited by interferon. Fish Shellfish Immunol.

[B9] McBeath AJ, Collet B, Paley R, Duraffour S, Aspehaug V, Biering E, Secombes CJ, Snow M (2006). Identification of an interferon antagonist protein encoded by segment 7 of infectious salmon anaemia virus. Virus Res.

[B10] Dannevig BH, Falk K, Krogsrud J (1993). Leucocytes from Atlantic salmon, *Salmo salar L.*, experimentally infected with infectious salmon anemia (ISA) exhibit an impaired response to mitogens. J Fish Dis.

[B11] Sokolskaja E, Luban J (2006). Cyclophilin, TRIM5, and innate immunity to HIV-1. Curr Opin Microbiol.

[B12] Towers GJ (2007). The control of viral infection by tripartite motif proteins and cyclophilin A. Retrovirology.

[B13] Krasnov A, Koskinen H, Pehkonen P, Rexroad CE, Afanasyev S, Molsa H (2005). Gene expression in the brain and kidney of rainbow trout in response to handling stress. BMC Genomics.

[B14] MacKenzie S, Montserrat N, Mas M, Acerete L, Tort L, Krasnov A, Goetz FW, Planas JV (2006). Bacterial lipopolysaccharide induces apoptosis in the trout ovary. Reprod Biol Endocrinol.

[B15] Munir K, Kibenge FS (2004). Detection of infectious salmon anaemia virus by real-time RT-PCR. J Virol Methods.

[B16] Mikalsen AB, Teig A, Helleman AL, Mjaaland S, Rimstad E (2001). Detection of infectious salmon anaemia virus (ISAV) by RT-PCR after cohabitant exposure in Atlantic salmon *Salmo salar*. Dis Aquat Organ.

[B17] He B (2006). Viruses, endoplasmic reticulum stress, and interferon responses. Cell Death Differ.

[B18] Liu KD, Gaffen SL, Goldsmith MA (1998). JAK/STAT signaling by cytokine receptors. Curr Opin Immunol.

[B19] Uddin S, Platanias LC (2004). Mechanisms of type-I interferon signal transduction. J Biochem Mol Biol.

[B20] Lu B (2006). The molecular mechanisms that control function and death of effector CD4+ T cells. Immunol Res.

[B21] Papa S, Zazzeroni F, Pham CG, Bubici C, Franzoso G (2004). Linking JNK signaling to NF-kappaB: a key to survival. J Cell Sci.

[B22] Liu FT (2005). Regulatory roles of galectins in the immune response. Int Arch Allergy Immunol.

[B23] Rabinovich GA, Gruppi A (2005). Galectins as immunoregulators during infectious processes: from microbial invasion to the resolution of the disease. Parasite Immunol.

[B24] Imaizumi T, Kumagai M, Sasaki N, Kurotaki H, Mori F, Seki M, Nishi N, Fujimoto K, Tanji K, Shibata T, Tamo W, Matsumiya T, Yoshida H, Cui XF, Takanashi S, Hanada K, Okumura K, Yagihashi S, Wakabayashi K, Nakamura T, Hirashima M, Satoh K (2002). Interferon-gamma stimulates the expression of galectin-9 in cultured human endothelial cells. J Leukoc Biol.

[B25] Imaizumi T, Yoshida H, Nishi N, Sashinami H, Nakamura T, Hirashima M, Ohyama C, Itoh K, Satoh K (2007). Double-stranded RNA induces galectin-9 in vascular endothelial cells: involvement of TLR3, PI3K, and IRF3 pathway. Glycobiology.

[B26] Pillai S, Baltimore D (1988). The omega and iota surrogate immunoglobulin light chains. Curr Top Microbiol Immunol.

[B27] Tangye SG, Nichols KE, Hare NJ, van de Weerdt BC (2003). Functional requirements for interactions between CD84 and Src homology 2 domain-containing proteins and their contribution to human T cell activation. J Immunol.

[B28] Chroboczek J, Gout E, Favier AL, Galinier R (2003). Novel partner proteins of adenovirus penton. Curr Top Microbiol Immunol.

[B29] Enjuanes L, Almazan F, Sola I, Zuniga S (2006). Biochemical aspects of coronavirus replication and virus-host interaction. Annu Rev Microbiol.

[B30] Gober MD, Wales SQ, Aurelian L (2005). Herpes simplex virus type 2 encodes a heat shock protein homologue with apoptosis regulatory functions. Front Biosci.

[B31] Mayer MP (2005). Recruitment of Hsp70 chaperones: a crucial part of viral survival strategies. Rev Physiol Biochem Pharmacol.

[B32] Riccioni G, Bucciarelli T, Mancini B, Di Ilio C, D'Orazio N (2007). Antileukotriene drugs: clinical application, effectiveness and safety. Curr Med Chem.

[B33] Steer SA, Corbett JA (2003). The role and regulation of COX-2 during viral infection. Viral Immunol.

[B34] Imai T (2006). Human carboxylesterase isozymes: catalytic properties and rational drug design. Drug Metab Pharmacokinet.

[B35] Hamm JK, el Jack AK, Pilch PF, Farmer SR (1999). Role of PPAR gamma in regulating adipocyte differentiation and insulin-responsive glucose uptake. Ann N Y Acad Sci.

[B36] Alam T, An MR, Papaconstantinou J (1992). Differential expression of three C/EBP isoforms in multiple tissues during the acute phase response. J Biol Chem.

[B37] Hu HM, Baer M, Williams SC, Johnson PF, Schwartz RC (1998). Redundancy of C/EBP alpha, -beta, and -delta in supporting the lipopolysaccharide-induced transcription of IL-6 and monocyte chemoattractant protein-1. J Immunol.

[B38] Jorgensen SM, Hetland DL, Press CM, Grimholt U, Gjoen T (2007). Effect of early infectious salmon anaemia virus (ISAV) infection on expression of MHC pathway genes and type I and II interferon in Atlantic salmon (*Salmo salar L.*) tissues. Fish Shellfish Immunol.

[B39] Jensen I, Robertsen B (2002). Effect of double-stranded RNA and interferon on the antiviral activity of Atlantic salmon cells against infectious salmon anemia virus and infectious pancreatic necrosis virus. Fish Shellfish Immunol.

[B40] Fernandez-Sesma A, Marukian S, Ebersole BJ, Kaminski D, Park MS, Yuen T, Sealfon SC, Garcia-Sastre A, Moran TM (2006). Influenza virus evades innate and adaptive immunity via the NS1 protein. J Virol.

[B41] Martin SA, Zou J, Houlihan DF, Secombes CJ (2007). Directional responses following recombinant cytokine stimulation of rainbow trout (*Oncorhynchus mykiss*) RTS-11 macrophage cells as revealed by transcriptome profiling. BMC Genomics.

[B42] Zou J, Mercier C, Koussounadis A, Secombes C (2007). Discovery of multiple beta-defensin like homologues in teleost fish. Mol Immunol.

[B43] O'Farrell C, Vaghefi N, Cantonnet M, Buteau B, Boudinot P, Benmansour A (2002). Survey of transcript expression in rainbow trout leukocytes reveals a major contribution of interferon-responsive genes in the early response to a rhabdovirus infection. J Virol.

[B44] Santoro MG (1996). Viral infection. Exs.

[B45] Glaser KB (1995). Regulation of phospholipase A2 enzymes: selective inhibitors and their pharmacological potential. Adv Pharmacol.

[B46] Greaves MW, Camp RD (1988). Prostaglandins, leukotrienes, phospholipase, platelet activating factor, and cytokines: an integrated approach to inflammation of human skin. Arch Dermatol Res.

[B47] Khanapure SP, Garvey DS, Janero DR, Letts LG (2007). Eicosanoids in inflammation: biosynthesis, pharmacology, and therapeutic frontiers. Curr Top Med Chem.

[B48] Janeway CA (2005). Immunobiology: the immune system in health and disease.

[B49] Moran TM, Park H, Fernandez-Sesma A, Schulman JL (1999). Th2 responses to inactivated influenza virus can Be converted to Th1 responses and facilitate recovery from heterosubtypic virus infection. J Infect Dis.

[B50] Graham MB, Braciale VL, Braciale TJ (1994). Influenza virus-specific CD4+ T helper type 2 T lymphocytes do not promote recovery from experimental virus infection. J Exp Med.

[B51] Kash JC, Tumpey TM, Proll SC, Carter V, Perwitasari O, Thomas MJ, Basler CF, Palese P, Taubenberger JK, Garcia-Sastre A, Swayne DE, Katze MG (2006). Genomic analysis of increased host immune and cell death responses induced by 1918 influenza virus. Nature.

[B52] Kobasa D, Jones SM, Shinya K, Kash JC, Copps J, Ebihara H, Hatta Y, Kim JH, Halfmann P, Hatta M, Feldmann F, Alimonti JB, Fernando L, Li Y, Katze MG, Feldmann H, Kawaoka Y (2007). Aberrant innate immune response in lethal infection of macaques with the 1918 influenza virus. Nature.

[B53] Rasmussen R, Meuer SWCNK (2001). Quantification on the LightCycler. Rapid Cycle Real-Time PCR.

[B54] Jorgensen SM, Kleveland EJ, Grimholt U, Gjoen T (2006). Validation of reference genes for real-time polymerase chain reaction studies in Atlantic salmon. Mar Biotechnol (NY).

[B55] Pfaffl MW (2001). A new mathematical model for relative quantification in real-time RT-PCR. Nucleic Acids Res.

[B56] Vandesompele J, De Preter K, Pattyn F, Poppe B, Van Roy N, De Paepe A, Speleman F (2002). Accurate normalization of real-time quantitative RT-PCR data by geometric averaging of multiple internal control genes. Genome Biol.

